# Cost-Effectiveness of Structural Health Monitoring in Aviation: A Literature Review

**DOI:** 10.3390/s25196146

**Published:** 2025-10-04

**Authors:** Pietro Ballarin, Giuseppe Sala, Alessandro Airoldi

**Affiliations:** Department of Aerospace Science and Technology, Politecnico di Milano, 20156 Milan, Italy; giuseppe.sala@polimi.it (G.S.); alessandro.airoldi@polimi.it (A.A.)

**Keywords:** structural health monitoring, aeronautical structures, economic impact assessment, fiber optic sensors, piezoelectric sensors, maintenance, production, composites

## Abstract

(1) Background: Structural Health Monitoring Systems (SHMSs) can reduce maintenance costs and aircraft downtime. However, their economic impact remains underexplored, particularly in cost–benefit terms. (2) Methods: This study conducted a targeted literature review on all the existing studies consisting of seventeen economic analyses of SHMS applications. Key features—such as SHMS type, structural material, vehicle type, integration stage, and cost elements—were classified to identify prevailing trends and gaps. (3) Results: The analysis revealed a predominance of piezoelectric-based SHMS applied to metallic fixed-wing aircraft, with limited attention to composite structures and e-VTOLs. Most studies focused on maintenance phase impacts, overlooking integration costs during manufacturing. Potential benefits like operational life extension, prognostic capabilities, and safety margin reduction were rarely explored, while critical drawbacks such as detection performance, reliability, and power consumption were underrepresented. Maintenance and fuel costs were the most frequently considered economic drivers; downtime costs were often neglected. (4) Conclusions: Although the majority of reviewed studies suggest a positive economic impact from SHMS implementation, significant gaps remain. Future research should address SHMS reliability, integration during early design stages, and applications to emerging aircraft like e-VTOLs to fully realize SHMS economic advantages.

## 1. Introduction

Structural integrity is a key concern in engineering, particularly in the aerospace sector, where failures can result in the loss of human lives [1]. At the same time, minimizing the weight of an aircraft is essential for market competitiveness [2]. Once the Maximum Takeoff Weight (MTOW) is established as a design parameter, maximizing passenger capacity often depends on reducing structural weight. This leads to the removal of material within the limits set by safety requirements [3]. To ensure safety, aircraft structures are designed to withstand expected operational loads. However, real-life conditions can differ significantly from the assumptions made during the design process. Furthermore, it has also been noted in the literature, especially when using pre-preg composite materials [4,5], that defects may be inadvertently introduced [6,7]. Additional damage may result from random events such as hail [8,9], bird strikes [10,11], and FOD, which can sometimes go undetected during routine inspections. Internal damage is especially critical, as it can seriously affect safety but is harder to detect due to limitations of current inspection technologies and restricted access for maintenance personnel. To maintain high safety standards, each aircraft follows a specific maintenance program [12,13,14], which includes detailed inspection schedules and procedures to repair or replace parts. These inspections usually require the disassembly of structural components, which are then examined using various Non-Destructive Testing (NDT) methods [15,16,17] such as tapping, radiography, thermography, and ultrasonic testing for composite parts, while eddy currents, liquid penetrant, and magnetic particle testing are used in metallic structural elements. Visual inspection is used instead in both types of structures. Although these techniques are effective, they increase aircraft downtime and require significant labor from engineers and technicians. In fact, maintenance alone can account for up to 27% of an aircraft’s total lifecycle cost [18].

A promising alternative is to use sensors permanently integrated into the structure to monitor its condition, potentially reducing maintenance costs while preserving safety [19]. Over the past 30 years, the implementation of Structural Health Monitoring Systems (SHMSs) in aerospace has been extensively studied [1,19,20,21,22,23,24,25]. SHMSs aim to continuously assess the condition of a structure in real time, ideally without human intervention. Their importance has grown, especially due to their potential in predicting failures before they occur. Despite the growing body of research validating SHMS performance and reliability [26,27,28,29,30], their integration into civil aviation remains cautious. It is important to note that some SHMS technologies, such as Comparative Vacuum Monitoring, have received FAA approval and are already in operational use by commercial airlines, notably on Delta’s B737 fleet, as well as in military and uncrewed aerial vehicles. These real-world applications highlight the increasing technological maturity and trust in SHMS for structural monitoring. However, despite these operational successes, the cost-effectiveness of SHMS adoption, defined as the net cost reduction over the life cycle of the aircraft, remains underrepresented in the literature.

Purchasing a new aircraft is just the beginning of a much larger financial investment [31,32]. Ownership involves ongoing operational expenditures, which are necessary to keep the fleet functional and generate revenue. According to [33], typical Direct Operating Costs include: (i) depreciation; (ii) insurance; (iii) fuel; (iv) crew expenses; (v) maintenance; and (vi) fees such as landing, navigation, and emissions. Assessing the economic viability of a new solution, such as SHMS, requires consideration of all these variables. However, many of these aspects are difficult to quantify precisely. This makes the evaluation of SHMS’s economic impact complex. Still, as outlined in Section 3, several studies have attempted to estimate their effect on aircraft lifecycle costs.

This review aims to address the growing need for a structured understanding of how SHMSs affect aircraft lifecycle costs. While technical aspects of SHMSs have been widely discussed in previous reviews [22,34,35,36], the economic dimension remains underexplored. To guide our analysis and clarify the scope, the following research questions were defined:

1. What types of cost-benefit evaluations have been performed for SHMSs in aerospace structures, and which economic variables are most commonly considered?
2. How do SHMS technologies differ in terms of their application contexts (e.g., structural materials, aircraft types, lifecycle stages), and how does this affect their assessed economic impact?
3. What are the major gaps and limitations in the existing economic studies, and where should future research focus to improve the realism and relevance of SHMS cost models?

These guiding questions provide a framework for selecting the reviewed literature, categorizing the case studies, and interpreting the economic models applied. They also help delineate the boundaries of this review—focused exclusively on SHMS for aircraft structural elements, excluding systems related to propulsion, avionics, or general onboard diagnostics.

It is important to recognize that the majority of real-world aviation incidents and technical failures are not caused by structural issues, but by system-level failures, such as those involving engines, avionics, landing gear, and hydraulic subsystems. Several large-scale analyses of aviation maintenance data and incident reports [37] confirm this pattern. While SHMS technologies for systems have matured significantly—ranging from engine health monitoring to prognostics for actuators and avionics—the current review intentionally focuses on airframe SHMSs, and more specifically on studies that quantitatively assess its economic impact. This focus is motivated by a documented gap in the literature: while the technological performance of structural SHMS has been widely studied [30,38], the economic case for their adoption remains fragmented and underdeveloped [18]. Airframe SHMS also faces unique challenges related to sensor embedding [39], certification, weight penalties [33], and interaction with traditional damage-tolerant design philosophies, which are not directly comparable to system-level SHMS. Therefore, this review aims to complement broader surveys of SHMS in aerospace [21,34,35,40] by systematically analyzing the cost-effectiveness literature specific to airframe structures. A broader review including system-level SHMSs would be valuable but is beyond the scope of this paper.

While SHM in aerospace has been widely studied for over two decades [1,19,21,22,23], most reviews have focused on sensor technologies, signal processing, or damage detection methods [22,34,35,36], as is shown in Table 1. In contrast, studies that provide quantitative economic assessments—evaluating lifecycle costs, maintenance savings, or return on investment for SHMSs in structural components—are relatively scarce. The first economic case study, to the best of the authors’ knowledge, was published in 2010 [41], and only 17 works meeting these criteria have been identified to date. Thus, this review fills a specific gap in the literature by systematically analyzing and classifying cost–benefit analyses of SHMS in aerospace structures, rather than SHM technologies in general.

The paper is structured as follows. Section 2 presents the methodology adopted to carry out this literature review. Section 3 discusses current maintenance strategies and the potential integration of SHMSs into the aircraft lifecycle. Section 4 presents a literature review and a summary of case studies. Section 5 analyzes these case studies in detail, highlighting the main variables and methodologies used to assess SHMS cost-effectiveness. In Section 6, the results obtained in the previous Section 5 are discussed. Finally, Section 7 presents the conclusions and key findings, emphasizing the benefits, limitations, and future research needed to enhance economic evaluations of SHMSs in aerospace contexts.

## 2. Methodology of Literature Selection

This review followed a targeted literature search approach with clearly defined inclusion and exclusion criteria to identify studies that quantitatively assess the economic impact of SHMS in aerospace structural applications.

### 2.1. Search Strategy

Searches were conducted in Scopus, Web of Science, and Google Scholar using combinations of the following keywords: (“Structural Health Monitoring” OR SHM OR SHMS) AND (aerospace OR aircraft OR rotorcraft OR fuselage OR wing) AND (economic OR cost-benefit OR lifecycle cost OR maintenance cost). Searches included literature published until early 2025.

### 2.2. Inclusion Criteria

- Studies must explicitly evaluate the economic impacts of SHMSs, either through cost-benefit analysis, lifecycle cost modeling, or direct operating cost comparisons.
- The SHMS must be applied to aircraft structural elements (e.g., fuselage, wing, and rotor blade).
- Studies must involve either civil or military aircraft, including fixed-wing, rotorcraft, or uncrewed aerial vehicles (UAVs).
- Peer-reviewed journal articles, conference papers, and reputable institutional reports were included.

### 2.3. Exclusion Criteria

- Studies focused solely on system-level monitoring (e.g., engines, avionics, hydraulic systems) were excluded.
- Technical papers describing SHMS technologies without any economic analysis were excluded.
- Studies lacking sufficient information to reconstruct or understand the cost assumptions were also excluded.

### 2.4. Selection Process

An initial pool of 74 papers was identified. Titles and abstracts were screened for relevance. After full-text review and application of the criteria above, 17 papers were selected for final analysis. Both studies based on modeling, using simulation or cost models, and empirical, incorporating real-world data or validation scenarios, were considered in this review. Both journal and conference papers were retained. We acknowledge the diversity in modeling assumptions across studies (e.g., SHMS performance levels, cost variables considered, discount rates), and this was reflected in our analysis of limitations and future research directions (see Section 6).

### 2.5. Rationale for Classification Dimensions

The classification dimensions used in this review (e.g., sensor type, structural material, lifecycle stage, maintenance strategy, SHMS integration method, cost variable types) were derived using a hybrid approach:

- **Inductive derivation**: During the full-text analysis of the 17 selected studies, recurring themes and analytical parameters were identified. For example, several papers made distinctions between Scheduled-SHM and Online-SHM strategies, or assessed the impact of SHMS on operational life extension. These patterns informed the development of key categorical variables (e.g., SHMS integration type, life cycle stage, performance assumptions).
- **Established frameworks and domain knowledge**: Other dimensions were grounded in prior literature and aerospace industry practices. For instance:
  – The Maintenance Steering Group-3 (MSG-3) [42] framework and the Condition-Based Maintenance (CBM) paradigm informed the categorization of maintenance strategies [43,44].
  – SHMS reliability dimensions such as Probability of Detection (POD) and Probability of False Alarm (PFA) are widely used in SHM validation and regulatory assessment [30,38,45].
  – Classifying cost drivers (e.g., maintenance labor, fuel, downtime) aligns with standard direct operating cost structures in aviation economics [33].

These classification dimensions serve two purposes: (i) to identify patterns and gaps in the literature regarding SHMS economic modeling, and (ii) to support comparability across studies with diverse scopes and assumptions. This mixed-method taxonomy supports a structured and objective synthesis of a relatively small but heterogeneous body of research.

## 3. Current Maintenance Procedures in Aviation and Integration of SHMSs

Today, aircraft structural maintenance is performed at fixed intervals under a methodology known as scheduled maintenance. These maintenance plans are developed following the MSG-3 approach [42], which reflects the collaboration of aircraft manufacturers, regulatory bodies, operators, and the Air Transport Association of America. The primary goal of MSG-3 is to establish tailored maintenance schedules that ensure a minimum acceptable level of safety for each specific aircraft model.

For metallic structures, maintenance intervals are determined based on a damage accumulation model, where cracks propagate over time under operational loads. Components must be repaired or replaced before these cracks reach a critical length. Inspection intervals are calculated by estimating the time it takes for the damage to reach this threshold and then applying a safety margin to ensure early detection [46].

In the case of composite structures, the most significant and hazardous failure mode is delamination [47,48]. As composites are relatively new compared to metallic materials in aerospace applications, a conservative maintenance philosophy is still applied. Regulations typically do not permit damage to grow under load, although in certain cases, limited damage growth is accepted [49]. Therefore, inspection intervals for composites are defined using a probabilistic method, based on estimating the probability of failure throughout the service life of the structure.

Scheduled maintenance is characterized by periodic inspections where components are examined or replaced if damage exceeds predetermined thresholds. This remains the standard strategy for maintaining aircraft and rotorcraft. Scheduled maintenance is organized into hierarchical levels based on inspection depth and frequency [50,51]:

- A-check: A light inspection, performed every 2–4 weeks. It includes checks of fluids, filters, and basic lubrication.
- B-check: Often incorporated into A-checks and thus rarely performed separately.
- C-check: Conducted every 12–20 months. It involves a more thorough inspection of systems and limited structural elements.
- D-check: The most extensive, performed every 6 to 12 years. The aircraft is taken out of service for several weeks. Paint and external panels are removed to allow deep structural inspection and component replacement, including internal parts.

The integration of SHMSs in aircraft maintenance is most relevant to C-checks and D-checks, as these involve structural rather than system-level assessments. Several studies in the literature have explored various strategies for incorporating SHMSs into aircraft maintenance frameworks. For clarity, this work distinguishes between Online and Scheduled-SHM. Online-SHM refers to systems that continuously monitor structural health during flight operations, whereas Scheduled-SHM describes systems interrogated by maintenance personnel during regular inspections. Both types fall under the broader category of CBM, as they initiate maintenance actions based on sensor-detected conditions. SHMSs enable a transition from traditional scheduled maintenance to CBM [40,44], where maintenance tasks are executed only when deemed necessary by the system. This can significantly reduce costs by improving aircraft availability and optimizing personnel allocation. Beyond damage detection, SHMSs can enable prognostic capabilities, allowing for the estimation of the remaining useful life of structural components [52,53,54]. This information forms the foundation of predictive maintenance, which shifts maintenance planning from fixed intervals to condition-based, data-driven decisions [55]. By accurately forecasting when a component will reach the end of its serviceable life, operators can reduce unplanned downtime, avoid premature replacement, and optimize the scheduling of inspections and repairs. Moreover, ’remaining useful life’ estimates contribute to spare parts inventory optimization, enabling maintenance facilities to stock only what is needed—when it is needed—thus lowering warehousing costs and avoiding both shortages and overstock. This prognostic insight also enhances fleet-level maintenance coordination, especially in large operations where asset availability is tightly linked to economic performance.

## 4. Overview of Economic Studies Presented in the Literature

As anticipated in the introductory section, since this work is specifically related to SHMSs, all the works including the monitoring of the aircraft systems (e.g., the engine, hydraulic plant, electric plant, and actuators) were not considered.

The economic impact assessment of SHMS has already been explored in the literature, particularly in the context of aircraft. For instance, in [18], the effect of a piezo-based SHMS on the metallic fuselage of a Boeing 737 was assessed under a Scheduled-SHM framework. The SHMS was intended for use by maintenance technicians during C-checks, which occur every 2800 flights and require the removal of certain non-structural components. The structural parts were assumed to remain intact, while the SHMS enabled automated inspections through ground-connected equipment to detect potential damage. The study found that while SHMS reduced aircraft downtime, the increased fuel consumption due to its weight resulted in higher operational costs. Another economic study regarding a metallic fuselage was presented in [56], where the authors compared four maintenance strategies: (i) scheduled maintenance; (ii) CBM using continuous onboard SHMS; (iii) scheduled-SHM; and (iv) CBM-skip. The latter two differ from CBM in that the actuators are not permanently onboard—the excitation is provided at intervals when the aircraft is on the ground. Scheduled-SHM mimics scheduled maintenance inspection intervals, whereas CBM-skip features more frequent SHMS interrogation. These two methods form hybrids between scheduled maintenance and CBM. It was found that all SHMS-based approaches lead to significant economic savings, with increased fuel costs being offset by substantial reductions in maintenance efforts. Other studies were presented on the economic impact of SHMSs on the aircraft’ fuselage [41,57]. For instance, in [41], the SHMS application to a metallic fuselage subjected to pressurization cycles was analyzed, accounting for system weight, performance, and aging. The primary cost drivers were increased fuel consumption and reduced manual inspection costs. Overall, an economic benefit was observed. Finally, in [57], CBM was evaluated for a metallic aircraft fuselage using an Online-SHM system. A comparison between scheduled maintenance and CBM was made, maintaining the same probability of a crack reaching a critical length. The case study involved a fuselage panel of a civil transport aircraft and showed that CBM offered a clear economic advantage. The authors also identified an optimal thickness that minimized total cost, balancing the benefits of extended crack growth time (from increased thickness) against the higher fuel cost due to added weight. Focusing on the integration of the sensors in an aircraft fuselage, the so-called Beginning Of Life (BOL), the authors in [58] built a cost model to investigate the costs of integrating piezoelectric sensors into a composite aircraft fuselage using a bottom-up methodology. This study focused on the manufacturing phase rather than maintenance and considered operational costs only in terms of weight increase, without accounting for potential economic benefits associated with CBM. The economic impact of piezo-based SHMS was extensively investigated in the literature in addition to those already presented. Indeed, the authors in [33] adopted the SHMS as a replacement for detailed visual inspections during A-checks and C-checks using piezoelectric sensors. An Online-SHM system was considered, where manual inspection was still required upon SHMS indication. The study noted a “snowball effect,” where increasing the number of sensors negatively affected aircraft performance due to added weight, eventually rendering SHMS economically disadvantageous. The same authors presented another study concerning the economic impact of piezo-based SHMSs [59]. The study focused on a composite structure with Online-SHM. The safety factor was reduced from 2.0 to 1.75, leading to estimated direct operating cost reductions between 2% and 5%, assuming equivalent performance and a 50% decrease in maintenance costs. In [60], a piezo-based SHMS was analyzed for Online-SHM on the metallic structure of an F-15. Three strategies were compared: (i) scheduled maintenance with inspections based on the single flight probability of failure; (ii) Online-SHM; and (iii) a mixed strategy—Online-SHM early in the aircraft’s life, followed by scheduled maintenance later. While Online-SHM yielded the best availability, the lowest ownership cost was achieved with scheduled maintenance. The authors in [61] did not implement any cost model, but they focused on the impact of piezo-based SHMS to raise allowable design limits and reduce structural mass in a composite aircraft. Under an Online-SHM assumption, the study showed economic gains in damage-tolerant designs, while structures governed by instability criteria did not benefit. A 5% weight reduction was achieved by decreasing thickness, even when including SHMS mass.

Some works presented in the literature evaluated the economic impact of fiber Bragg-grating (FBG)-based SHMSs [62,63,64]. For example, in [62], the SHMS application on an Airbus A320 was examined using both ultrasonic and FBG sensors, replacing detailed visual inspection and general visual inspections. An Online-SHM approach was assumed, and the authors developed a weight and a power model for the SHMS. The results showed that, despite the increase in weight and power consumption of the SHMS, the latter led to economic benefits. The work presented in [63] was aimed at assessing the economic impact of FBG-based SHMS on the BOL stage of life cycle, during the development of a new helicopter rotor blade. In this stage, the sensors performed multiple functions: (i) temperature monitoring during curing for quality assurance; (ii) strain measurement during certification tests; and (iii) strain monitoring during load survey activities. These uses helped justify the SHMS cost at early stages. In [64], the authors assessed the economic impact of the same SHMS on the maintenance of a composite helicopter tail rotor blade. The SHMS replaced tap hammer inspections. However, the cost savings were outweighed by the higher cost of the blade as a spare part, increased repair complexity, and false alarms. The authors recommended limiting SHMS application to components less exposed to impact risks.

Some authors considered a probabilistic approach to assess the economic impact of the SHMS [65,66,67]. Indeed, in [65], the SHMS was exploited in the context of D-checks for a civil aircraft, a maintenance phase comprising five steps: component removal, inspection, defect repair, painting, and final checks. A Scheduled-SHM scenario was modeled, and 100 D-checks were simulated, introducing statistics and using Arena software. The SHMS activities rarely aligned with the critical path, offering limited benefit. The author concluded that to realize SHMS advantages, aircraft should be re-designed—especially components that are hard to access, such as those beneath fuselage gantries. In [66], the authors studied SHMS impacts in a Scheduled-SHM context for metallic aircraft. They emphasized that current inspection intervals represent a trade-off between safety and cost: more frequent inspections raise costs, while fewer inspections reduce safety. SHMSs alter this balance by lowering inspection costs. However, the occurrence of false alarms, which require traditional inspection follow-ups, was also considered. In [67], the impact of SHMS on composite structures was analyzed using probabilistic damage tolerance methods and considering the synchronization of the SHMS-related tasks with the scheduled maintenance. Four strategies were assessed: (i) CBM without SHMS; (ii) Scheduled-SHM; (iii) Scheduled-SHM with tenfold interrogation frequency; and (iv) Online-SHM. The Online-SHM strategy resulted in the lowest total maintenance cost. In [68], the life cycle cost of a composite stiffened panel (part of the F-18 upper wing skin) was optimized. Costs included both traditional scheduled inspections and alarms triggered by the SHMS, which could result from real damage or false positives. The study concluded that the SHMS threshold—used to classify the structure as pristine or damaged—had a strong influence on life cycle cost and should be carefully selected.

The work presented in [69] considered the possibility of monitoring the loads: the authors explored the use of load monitoring to extend aircraft operational life. Fatigue indices were created for fuselage and wing structures using flight data such as distance, passenger count, and altitude to derive load spectra and reduce the uncertainty in the estimation of the remaining useful life.

## 5. Literature Analysis and Classification of Economic Studies

In this section, a comprehensive literature classification is carried out to analyze the integration of SHMSs throughout the aircraft lifecycle. The review focuses on the characteristics of SHMSs, the types of structures analyzed, and the variables considered in economic assessments. The goal is to identify research gaps and underscore the need for further scientific investigation. Only case studies specifically related to aircraft were included, while studies providing general insight into SHMS economics were not considered; furthermore, only case studies specifically related to the monitoring of structural elements were taken into consideration; therefore, studies considering the monitoring of the aircraft systems were out of the scope of this work. A total of 17 papers, the ones presented in the previous section, were analyzed.

This section is organized into two subsections: (i) Section 5.1 presents the main features that characterize the studies of the literature, such as, for example, the type of SHMS considered in the economic evaluations, the integration of the SHMS into the maintenance process and the final economic evaluation results; (ii) Section 5.2 is specifically related to the investigation of the main variables considered in the cost models adopted in the economic evaluation of the SHMS; (iii) Section 5.3 focuses on specific modeling details considered only by few studies; (iv) Section 5.4 analyses the interactions between the main selected features.

### 5.1. Main Features of the Economic Case Studies

**Figure 1:**
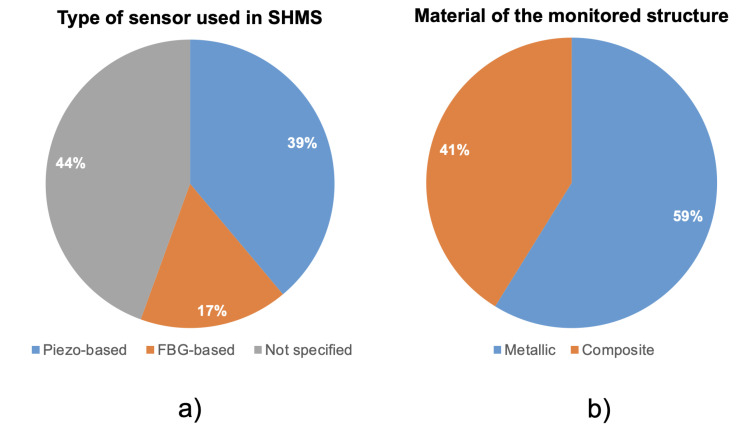
(**a**) Classification about the type of sensors adopted by the SHMS; (**b**) classification about the material of the monitored structure.

**Figure 2:**
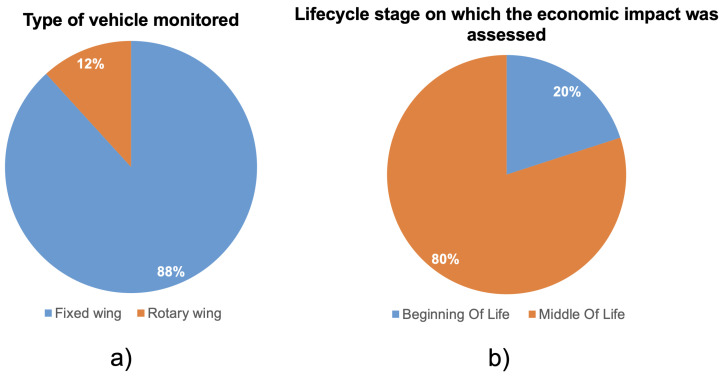
(**a**) Classification of the type of vehicle on which the SHMS was considered to be applied; (**b**) classification of the life cycle stages on which the cost-benefit assessment was performed.

**Figure 3:**
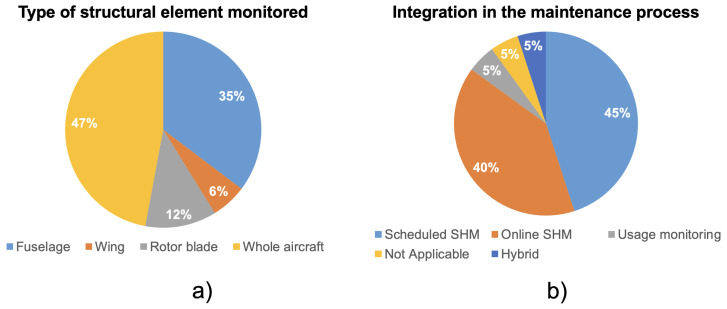
(**a**) Classification of the type of structural element on which the SHMS was considered to be applied; (**b**) classification of the integration in the maintenance process of the monitoring system.

**Figure 4:**
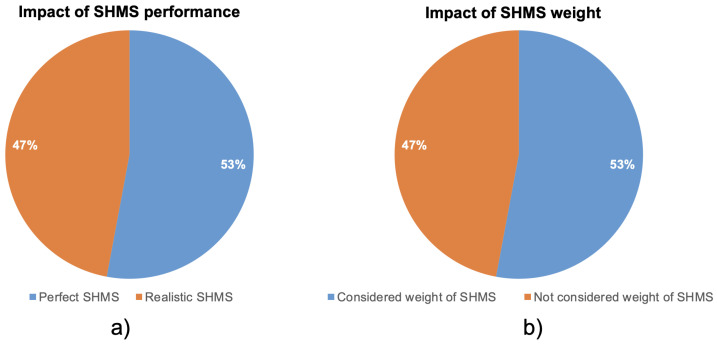
(**a**) Percentage of studies considering the performance of the SHMS in the economic evaluation; (**b**) percentage of studies considering the effects of the weight of the SHMS in the cost-benefit analysis.

In this section, the economic studies were classified on the basis of their main features, concerning the role and the application of the SHMS on the aircraft. The following features were analyzed:

- **Type of sensor used in the SHMS**: Various sensor types are employed to monitor structural integrity. The most common are piezoelectric sensors [27,70,71,72], FBG sensors [28,73,74,75], and Comparative Vacuum (CV) sensors [76,77,78]. However, due to the absence of case studies involving CV sensors, they were excluded. For some studies, the sensor type was unspecified. Thus, SHMS sensors classification was organized into: Piezo-based, FBG-based, and Not specified. However, most of the unspecified cases involved metallic structures, suggesting an implicit reliance on piezoelectric technology, which is standard in such applications. Thus, the actual prevalence of piezo-based SHMSs may be underreported. The study in [62] evaluated both Piezo and FBG-based SHMS; therefore, it is listed under both categories. In Figure 1a, the partition about the type of sensors adopted by the SHMS can be observed. It can be seen that the majority of the studies do not explicitly mention the type of SHMS taken into consideration. While 39% of them consider an SHMS based on piezo, the remaining 17% of the studies consider an FBG-based SHMS. One of the most striking observations is the widespread inconsistency in technical specificity. For example, although sensor technology is central to SHMS costs and capabilities, nearly half of the studies do not specify the type of sensor used. This raises questions about the robustness of the underlying cost models: how can a study justify an economic outcome without anchoring performance and integration cost to a known sensor type? It suggests that in many cases, economic modeling may be driven by high-level assumptions rather than grounded technical parameters.
- **Material of the monitored structure**: Given the increasing use of composites in aerospace, SHMS applications in such materials—typically governed by no-growth or slow-growth damage philosophies—warrant special attention. Hence, the studies were classified as involving either metallic or composite materials. In Figure 1b, the classification of the material of the monitored structure can be observed. It can be noted that the majority of the studies, 59% focused on metallic structures, while the remaining focused on composite structures. Although composite materials represent the future of aviation, it is notable that only a small portion of the studies focus on them. This may be due to the fact that studies on metallic structures tend to be older, whereas those addressing composites have been published more recently.
- **Type of vehicle monitored**: While the majority of the reviewed literature focuses on fixed-wing aircraft, only a small fraction (12%) addresses rotary-wing aircraft (Figure 2a). This imbalance is not merely descriptive—it suggests a potential research gap. Rotary-wing aircraft, such as helicopters, often operate under severe dynamic loads and are subject to higher vibration levels, which make them strong candidates for SHMS implementation. Moreover, differently from fixed-wing aircraft, their mission profile may change significantly from one helicopter to another, thus further motivating the adoption of SHMS. Their underrepresentation in the economic literature may indicate challenges related to data availability, complexity of modeling, or lower commercial prioritization. This trend reveals that the current body of economic evaluations may be disproportionately shaped by platform accessibility rather than operational criticality—an insight that should inform future research targeting high-need segments.

- **Lifecycle stage on which the economic impact was assessed**: The lifecycle of an aircraft is commonly divided into three main phases: BOL, Middle of Life (MOL), and End of Life (EOL) [79,80,81]. BOL encompasses manufacturing and delivery; MOL covers operational use and maintenance; EOL relates to decommissioning. Since SHMSs primarily influence the design, operation, and maintenance of the aircraft, the literature reviewed here focuses on the BOL and MOL phases.
  As shown in Figure 2b, a large majority of studies (approximately 80%) focus exclusively on the MOL, while only 20% explicitly address BOL considerations. This strong imbalance highlights a reactive trend in current SHMS economic modeling: the systems are often evaluated for their impact on existing maintenance procedures rather than as part of a broader design strategy. Only a few studies—e.g., [18,41,58]—attempt to assess SHMS across both stages. The underrepresentation of BOL-phase analysis reveals a significant missed opportunity. Early lifecycle integration is essential for realizing many of SHMS’s long-term benefits, such as structural weight optimization, wiring simplification, and platform-wide monitoring capability. By neglecting BOL, many studies may underestimate the total value or overlook critical trade-offs relevant to aircraft designers. This suggests a need for more holistic approaches that evaluate SHMSs not just as an operational enhancement, but as a strategic design investment.
- **Type of structural element monitored**: The scope of SHMS application varies significantly across studies. While some focus on individual structural components—such as the fuselage, wing, or rotor blade—others evaluate SHMS at the level of the entire aircraft. As shown in Figure 3a, the majority of studies take a whole-aircraft approach, while 35% focus on the fuselage, and only 6% consider the wing in isolation. This distribution reveals important trends. The prevalence of whole-aircraft modeling suggests an ambition to evaluate SHMS at a fleet or system level, potentially capturing broader operational benefits such as maintenance scheduling and aircraft availability. However, this approach may also reflect modeling simplifications, where detailed structural behaviors are abstracted into high-level assumptions. In contrast, component-specific studies—particularly those focused on the fuselage—tend to adopt more granular modeling, often incorporating material-specific damage mechanisms or localized inspection strategies. The underrepresentation of wing-focused studies is notable, given that wings are critical load-bearing structures and frequent sites of fatigue-related damage. Their relative absence in the economic literature may be due to the complexity of wing SHMS integration, aerodynamic sensitivities, or limited access for sensor installation. This imbalance suggests that current economic evaluations may be driven more by modeling feasibility than by structural criticality, and that future work should more systematically align SHMS scope with real-world failure distribution data.

- **Integration in the maintenance process**: The way SHMSs are integrated into the maintenance process significantly affects both their technical utility and economic impact. Systems may offer real-time (Online) monitoring, enabling continuous structural assessment during flight, or they may rely on scheduled interrogation, where data is extracted periodically during ground inspections. A hybrid approach combining the traditional inspections with the Online SHMS monitoring was also considered. A fourth category, usage monitoring, refers to SHMSs designed primarily to track operational loads throughout the aircraft’s life, rather than detect specific damage. For studies focused exclusively on early design stages (BOL), a Not Applicable category is included, as maintenance integration is not yet relevant. Some studies—such as [56,67]—consider multiple integration strategies and are thus represented in more than one category. As shown in Figure 3b, 45% of the reviewed studies evaluate Scheduled SHM, while 40% consider Online SHM, and only 5% investigate load monitoring capabilities and the combination of different maintenance strategies (hybrid approach). This distribution highlights a near-even split between the two dominant strategies, but the underutilization of usage monitoring is striking, especially given its relevance to fatigue tracking and mission-specific maintenance planning. The prominence of Scheduled SHM may reflect its compatibility with existing maintenance frameworks, making it easier to integrate without disrupting current operations. In contrast, Online SHM requires more substantial architectural integration (e.g., onboard processing, real-time data handling), but offers the greatest potential for cost savings through early anomaly detection and reduced downtime. Interestingly, preliminary analysis suggests that studies evaluating Online SHM are more likely to report positive economic outcomes, while Scheduled SHM results are more mixed. This could indicate that continuous monitoring better supports predictive maintenance strategies, or that economic models assume more aggressive benefits from online systems. However, without standardized evaluation frameworks, such conclusions remain tentative. Still, the data suggest a strategic shift toward online architectures, reflecting the broader aerospace trend toward condition-based and predictive maintenance paradigms.

- **Impact of SHMS performance**: Given the safety-critical role of SHMS, assumptions regarding their detection performance are central to any economic or operational analysis [38]. Key performance indicators include the POD and PFA [30,82], which directly influence maintenance decisions, inspection deferrals, and ultimately, lifecycle costs.
  In the reviewed literature, studies diverge significantly in how they model SHMS performance. Some adopt idealized assumptions, treating SHMS as having 100% POD and 0% PFA, thus enabling perfect damage detection and no false positives. Others incorporate realistic POD curves, acknowledging that detection is probabilistic and may vary based on damage size, sensor type, or location. In our classification, studies that model a realistic POD—regardless of whether they assume a nonzero PFA—are grouped under “realistic SHMS.”
  As shown in Figure 4a, 47% of the studies incorporate realistic performance assumptions, while the majority assume perfect SHMS behavior. This pattern reveals a tendency in the field to prioritize model simplicity over realism—potentially overestimating the value and reliability of SHMS. Assuming perfect detection capabilities removes the need to address false alarms, maintenance delays, or conditional inspection strategies, all of which are critical for actual implementation.
  Notably, this modeling choice can significantly influence economic outcomes. Several studies that incorporate realistic POD models still report positive cost-effectiveness, suggesting that SHMS can be economically viable even with imperfect detection. This underscores the robustness of certain system configurations, but also highlights a methodological inconsistency: there is no standardized threshold or benchmark for what constitutes “acceptable” SHMS performance in economic terms.
  The widespread use of perfect-performance assumptions likely stems from either lack of validated sensor performance data or an intent to establish best-case upper bounds. However, for SHMS to be integrated into safety-critical environments like commercial aviation, future studies must move beyond idealization and evaluate how performance uncertainty affects decision-making, maintenance intervals, and risk profiles.

- **Impact of SHMS weight**: The additional weight introduced by SHMS—whether from sensors, wiring, data acquisition units, or power sources—can have a substantial impact on aircraft performance. When MTOW limits are fixed, any added mass can either reduce payload capacity or increase fuel consumption over the aircraft’s lifecycle, thus directly influencing economic viability.
  In our review, studies were classified based on whether they explicitly included SHMS weight in their economic evaluation. As shown in Figure 4b, 53% of the studies accounted for the effects of added mass, while the remaining 47% omitted this factor altogether. This nearly even split highlights a key inconsistency in cost modeling practices: while over half of the studies attempt to reflect real-world integration constraints, a significant portion still rely on massless or abstract SHMS models.
  The omission of weight considerations can bias economic conclusions, as it disregards one of the most tangible trade-offs in SHMS adoption. Even modest increases in structural weight can result in meaningful lifecycle fuel penalties, especially in fuel-sensitive missions such as long-haul commercial flights or high-frequency regional operations. Interestingly, some of the studies that do model SHMS weight still report positive economic outcomes, suggesting that weight penalties can be mitigated or outweighed by benefits such as inspection cost reduction, increased aircraft availability, or reduced unscheduled maintenance. However, this finding also raises questions about how weight-related costs are calculated—and whether all studies use consistent assumptions regarding fuel burn sensitivity, mission profiles, or integration architectures.
  Overall, the treatment of SHMS weight in the literature remains fragmented, revealing a need for standardized modeling approaches that transparently incorporate mass-related penalties. Without this, comparisons between studies—and reliable conclusions about the cost-effectiveness of SHMS—remain difficult to make.

### 5.2. Modeling Details Considered by Few Studies

This subsection was introduced to point out some modeling details that were considered only by a reduced number of studies, namely: the aging of the SHMS, the potential of the SHMS to extend the operational life of the structural element, the power required by the SHMS, and the possibility of reducing the structural safety margins. Each of these modeling details is hereby discussed.

**Figure 5:**
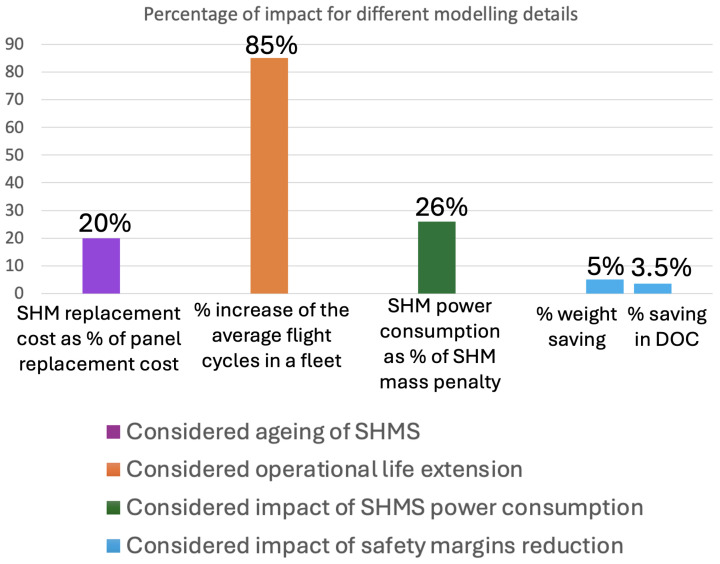
Percentage of impact for different modeling details. Each column indicates a study addressing a specific modeling detail.

- **Impact of SHMS aging**: Like all onboard systems, SHMSs are subject to aging-related degradation, which may affect their reliability, performance, and maintenance requirements over time. Components such as sensors, adhesives, and connectors can experience environmentally induced deterioration (e.g., thermal cycling, humidity exposure, vibration fatigue), potentially leading to increased false alarms, signal drift, or outright system failure. For example, studies such as [83,84] have shown that fiber FBG sensors may suffer from moisture-induced wavelength shifts, which compromise long-term measurement accuracy.
  Despite the relevance of this phenomenon, only one study in the reviewed literature explicitly considers SHMS aging, and the cost to substitute the monitoring system was estimated to be 20% of the cost to replace the entire aircraft’s panel, as can be see in Figure 5. The fact that only one study considers this aspect suggests that the durability and lifecycle reliability of SHMS remain underexplored dimensions in economic impact assessments. Most models appear to assume time-invariant system performance, thereby overlooking costs associated with recalibration, replacement, or performance degradation over time.
  This omission is concerning, as it implies that some positive economic conclusions may be overly optimistic—particularly for systems expected to operate over long service intervals or across high-cycle aircraft. Ignoring aging effects could underestimate maintenance costs or overstate availability improvements, especially in harsh operational environments (e.g., maritime patrol, tropical climates, or high-altitude operations).
  Moreover, the failure to model SHMS aging stands in contrast to the broader trend in aircraft lifecycle modeling, where component aging and degradation are routinely included for engines, avionics, and other critical systems. Extending such treatment to SHMSs would improve model realism and support more robust cost-benefit conclusions. As SHMS adoption scales toward full-aircraft coverage and long-duration platforms (e.g., commercial airliners, military transports), integrating aging behavior into economic models will become essential.
  In short, the lack of attention to SHMS aging represents a clear methodological gap in the literature and an opportunity for future work to provide more grounded, lifecycle-aware assessments of SHMS value.
- **Impact of operational life extension**: In conventional aircraft design, structural components are retired conservatively, based on worst-case assumptions about loading and fatigue accumulation. This approach ensures safety but often results in components being withdrawn well before their true end-of-life. One of the key promises of SHMSs is the ability to enable operational life extension by providing real-time or cumulative insights into structural health, allowing operators to defer replacement or inspection without compromising safety [69,85].
  Despite this potentially high-value benefit, our review reveals that only one study explicitly incorporates life extension into its economic evaluation, and the authors estimated a potential extension of 85% of the average flight, as reported in Figure 5. This omission is striking, especially considering that extended usage of structural elements could offer substantial cost savings in military, cargo, or legacy fleet applications—where extending airframe life is a strategic priority.
  The underrepresentation of this factor suggests that most existing cost models are conservatively framed, perhaps due to the difficulty of quantifying life extension benefits or a lack of long-term field data. Alternatively, it may reflect the risk-averse culture of aviation certification, where structural design lives are tightly regulated and changes to life limits require substantial validation.
  Nonetheless, failing to consider operational life extension understates the full economic potential of SHMS. Accurate structural tracking—especially through fatigue-prone areas—can allow for condition-based retirement, reducing unnecessary maintenance, deferrals, and parts replacement. For airlines and operators with high utilization rates, even modest life extensions could translate into millions in cost avoidance over a fleet’s operational horizon.
  Future cost-effectiveness models would benefit from explicitly incorporating probabilistic life extension scenarios, grounded in validated sensor data, to more fully capture SHMS’s potential value. This is particularly relevant as the industry moves toward digital twin frameworks and predictive maintenance ecosystems, where aircraft-specific structural histories can support tailored life management strategies.

- **Impact of SHMS power consumption**: Beyond structural weight, an SHMS imposes an electrical power demand that can subtly but meaningfully impact aircraft efficiency. Since onboard electricity is typically generated by the engines—either directly or via auxiliary power units—any additional power draw translates into increased fuel burn, particularly on long-haul flights or for aircraft operating in power-sensitive environments.
  As shown in Figure 5, only one of the reviewed studies considered this factor, and estimated a contribution of the cost related to the SHMS energy supply to be more or less 26% of the cost induced by the mass of the SHMS. This omission suggests that energy use is largely neglected in current cost-benefit frameworks, despite being an essential contributor to operational cost—especially as SHMS architectures become more complex and sensor-dense.
  This trend may stem from the relatively small magnitude of SHMS power draw per flight, leading some researchers to treat it as negligible. However, when considered across thousands of flight hours, the cumulative energy cost—especially for continuously operating systems such as Online SHM—can become non-trivial. Moreover, aircraft with more electric architectures will be even more sensitive to power distribution trade-offs, making this an increasingly relevant variable.
  The failure to consider power consumption also raises methodological concerns, as it introduces an optimistic bias in systems that offer real-time monitoring but at a continuous energy cost. Without accounting for this dimension, economic evaluations may overestimate the net benefit of such systems—particularly in marginal cases where lifecycle savings are small.
  Moving forward, future SHMS economic assessments should treat energy demand not as an optional parameter but as an integrated part of the system-level trade-off analysis, especially as aviation moves toward electrified and hybrid propulsion systems, where electrical efficiency becomes mission-critical.
- **Impact of safety margins reduction**: One of the most transformative potentials of SHMS lies in their ability to enable reduced structural safety margins without compromising airworthiness. Traditionally, aircraft structures are designed with conservative safety factors to account for uncertainty in loading, damage accumulation, and inspection intervals. However, with reliable real-time or near-real-time damage detection, SHMS could allow for higher allowable damage tolerance, faster crack growth acceptance, or thinner structures, ultimately reducing structural weight and improving overall efficiency. As shown in Figure 5, only two studies considered this potential: one estimates a potential weight saving of 5%, while the other estimates a potential saving in the direct operational costs of 3.5%. This under-representation suggests a significant missed opportunity: while safety margin reduction could deliver some of the greatest lifecycle cost savings through structural mass reduction and fuel efficiency gains, it remains largely unquantified in current models.
  The omission may be due to several factors. First, certification frameworks are currently built around conservative structural design assumptions, and reducing margins would require substantial regulatory changes and validation of SHMS reliability. Second, many economic evaluations focus on retrofitting SHMS into existing platforms, where redesign is not feasible, rather than on new aircraft programs where SHMS-informed structural optimization could be realistically pursued.
  Nonetheless, studies that do consider safety margin reduction demonstrate its potential to amplify the economic value of SHMS beyond inspection or maintenance savings alone. Incorporating this factor can shift the role of SHMS from a reactive maintenance tool to a design enabler, justifying more integrated, lightweight, and mission-tailored structures.
  As the industry evolves toward digital design, digital twins, and certification by simulation, SHMS has the potential to become a key enabler of more agile and efficient aircraft design. Future economic models should aim to integrate this dimension, not just as an assumption, but as a scenario-based variable, to better capture the full design-space benefits of SHMS adoption.

### 5.3. Evaluation of SHMS Cost-Effectiveness

A key dimension of this review is the assessment of whether SHMSs have been found to be economically viable in the existing literature. To capture the range of conclusions, studies were classified into three categories:

- **Positive**, indicating a favorable cost-benefit outcome,
- **Negative**, indicating that an SHMS increases lifecycle costs, and
- **Parametric**, where the study presents a sensitivity-based model without committing to a definitive conclusion (e.g., outcomes depend on system parameters or mission profiles).

**Figure 6:**
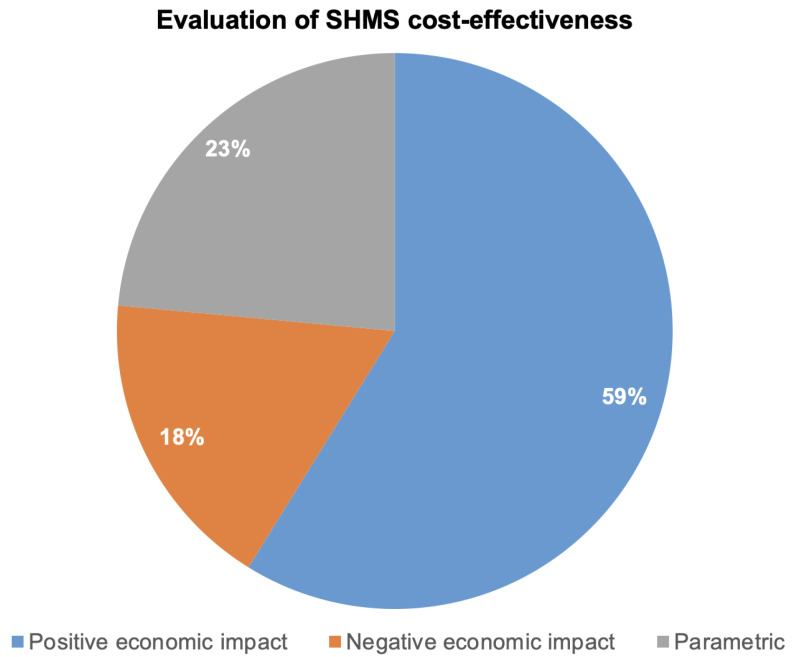
Results about the final economic evaluation of the case studies presented in the literature.

As illustrated in Figure 6, 59% of the studies report a positive economic impact, suggesting that SHMSs can improve cost-efficiency when applied under the right conditions. These benefits often stem from reduced inspection frequency, improved aircraft availability, or longer service intervals. However, this majority should not be over-interpreted as a universal endorsement. Many of these studies rely on optimistic assumptions regarding sensor reliability, integration costs, or weight penalties.

On the other end of the spectrum, 18% of studies conclude that SHMS has a negative economic impact, usually driven by factors such as added weight, system complexity, or the cost of integration exceeding the anticipated savings. These cases are particularly instructive as they highlight boundary conditions where SHMS may not yet be justifiable—such as for smaller platforms, short-duration missions, or low-utilization aircraft.

The remaining 23% of studies provide parametric models, often framed as design tools rather than conclusive evaluations. These models explore a range of scenarios based on variables like sensor cost, inspection intervals, or failure probabilities. While non-committal, they are methodologically valuable, as they offer flexible frameworks for evaluating SHMSs under diverse assumptions, and can guide stakeholders in making platform- or mission-specific decisions.

Taken together, this distribution indicates that while the literature leans toward economic optimism, there remains a significant degree of uncertainty. The diversity of assumptions, model structures, and performance estimates suggests that SHMS cost-effectiveness is highly context-dependent. A clear implication is the need for future studies to adopt transparent sensitivity analyses and scenario-based evaluations, ensuring that conclusions are robust to changes in design, operational profile, or technology maturity.

In Table 2, the main features of each reviewed article are summarized.

### 5.4. Cost Models Adopted in the Economic Assessment

To assess the economic impact of SHMS, each study integrates a specific set of cost variables into its model. These variables reflect both system costs (e.g., sensors, integration) and operational costs (e.g., fuel, downtime). For consistency, the terminology used across the literature was normalized to account for naming variations among different authors. The consolidated set includes:

- Cost of maintenance manpower,
- Cost of sensors,
- Cost of manpower for sensor integration,
- Cost of fuel,
- Cost of downtime,
- Cost of charges (e.g., airport or airspace fees),
- Cost of crew,
- Cost of passenger tickets,
- Cost of spare parts.

**Figure 7:**
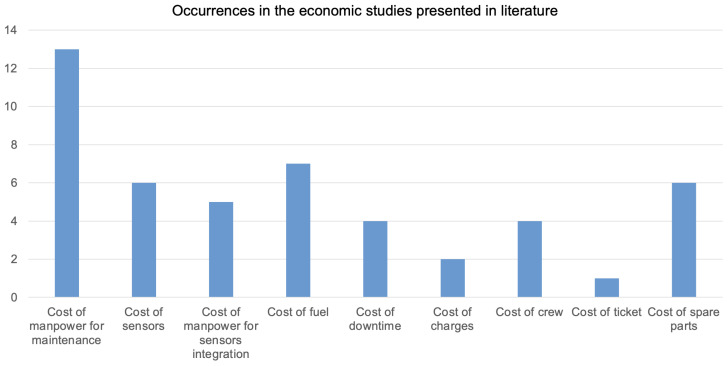
Occurrences of the variables adopted in the literature to assess the economic impact of the SHMS.

As illustrated in Figure 7, there is a strong bias toward a limited subset of these variables. The cost of maintenance manpower appears most frequently, used in 13 studies, followed by fuel cost in 7 studies, and both sensor cost and spare parts cost in 6 studies each. This prioritization reflects the areas where SHMS is expected to deliver the most tangible savings: reducing manual inspections, minimizing fuel penalties, and lowering unplanned part replacements.

However, the uneven adoption of cost factors also reveals a degree of inconsistency across studies. Variables such as downtime cost, crew cost, or integration labor are included sporadically, despite their clear relevance to SHMS operation. More strikingly, only one study—[18]—incorporates ticket cost, which could be particularly important when modeling the impact of SHMSs on commercial passenger aviation where flight delays or groundings translate directly into revenue loss or compensation claims.

The omission of certain variables in many studies suggests that cost models are often incomplete, leading to under- or overestimation of the true economic value of SHMS. Furthermore, the reliance on study-specific assumptions for costs (e.g., hourly rates, fuel price, part replacement intervals) makes comparisons across studies difficult. Without standardized cost categories or normalization procedures, the field risks drawing conclusions based on inconsistent modeling scopes.

Future work should aim to develop comprehensive and transparent cost frameworks, ideally aligned with operational profiles (e.g., commercial vs. military, long-haul vs. short-haul) and system maturity levels. This would improve both comparability and credibility of economic impact assessments and enable more confident decision-making for SHMS adoption.

### 5.5. Cross-Comparison Insights

It is interesting to note that the consideration of SHMS weight does not appear to systematically worsen economic outcomes. In fact, nine studies explicitly modeled the added mass of SHMS, and of those, six studies (66.7%) still reported a positive economic evaluation [41,56,57,59,61,62]. The remaining three were evenly split between parametric (2 studies; [33,58]) and negative (1 study; [18]) outcomes. This trend is counterintuitive, as the introduction of additional weight would typically be expected to negatively affect fuel consumption, payload efficiency, and ultimately lifecycle cost. These results suggest that some models may either undervalue the fuel penalty or assume that operational benefits (e.g., inspection deferral) sufficiently outweigh weight-related drawbacks. Another counterintuitive trend is the impact of SHMS performance modeling. Eight studies modeled real or realistic performance of the SHMS (as opposed to assuming perfect detection). Among these, six studies (75%) still reported positive outcomes [41,56,57,60,66,67]. Only one resulted in negative evaluations [64], and one was parametric [68]. This is unexpected because accounting for imperfect detection capability would normally degrade the perceived reliability and cost-benefit of SHMS. This discrepancy may again point to variation in the cost modeling assumptions, especially regarding detection thresholds, false alarms, and their operational consequences. These counterintuitive findings—that modeling weight or realistic performance does not necessarily lead to more negative outcomes—highlight a likely inconsistency in modeling assumptions and input data across the reviewed literature. Some studies may have accounted for benefits more comprehensively (e.g., inspection frequency reduction, increased aircraft availability), while others may have emphasized direct costs.

Studies that considered the whole aircraft as the monitored system—rather than focusing on a specific component such as the fuselage—were generally more likely to report a positive economic impact. Specifically, eight studies focused on whole aircraft SHMS deployment, and six of these concluded with a positive cost-effectiveness outcome [59,61,62,66,67,69]. Only one study among these reported a negative outcome [65], with two others showing parametric or inconclusive results. This trend may reflect either modeling optimism in aircraft-level SHMS applications or the assumed scale of benefits when fleet-level impact is considered. The maintenance integration strategy appears to influence outcomes: Online SHMS strategies are more likely to be associated with positive evaluations. Among the six studies using Online SHMS, four reported positive results [59,60,61,62], while two were parametric. In contrast, Scheduled SHMS integration led to positive outcomes in only three of seven studies [41,57,66], and negative or parametric in the others. This suggests that Online SHMS may be perceived as more effective in avoiding unscheduled maintenance or maximizing operational availability. Finally, it is interesting to observe a pattern between the monitored component and the assumed SHMS performance. Most studies addressing the whole aircraft as the structural scope assumed a perfect or ideal SHMS (e.g., [33,59,61,62,65,69]), while those focusing on the fuselage were more likely to model realistic performance (e.g., [41,56,57,60]). This could reflect the complexity of modeling detection behavior across larger structures, or the adoption of more conservative assumptions when addressing highly regulated, critical components like the fuselage. Among the six studies specifying piezoelectric sensors, three reported positive economic outcomes, two were parametric, and one was negative. These results point to a generally favorable view of piezo-based SHMSs, likely due to its maturity and lower integration costs. In contrast, the few studies involving FBG sensors show more conservative results, with one reporting a negative outcome due to realistic cost modeling [64], and one hybrid study (piezo + FBG) reporting a positive outcome [62]. However, this observation requires other FBG-related case studies to be validated and to find a general trend. Importantly, Figure 4a shows that 53% of the studies assumed perfect SHMS performance (100% POD, 0% PFA), while only 47% modeled realistic detection. This simplification weakens conclusion reliability, as performance parameters directly affect maintenance outcomes and false alarms. The frequent omission of such factors, particularly in studies reporting positive impacts, reflects a methodological gap that future work should address by incorporating sensor type, structural material, and detection reliability more explicitly.

## 6. Discussion

This section is structured into three subsections: the first provides an analysis and critical discussion of the review findings presented earlier; the second outlines future research directions; and the third addresses the limitations of this study.

### 6.1. Analysis of the Literature

This subsection exposes the assumptions, simplifications, and biases that currently shape perceptions of cost-effectiveness. While 59% of studies conclude that SHMS implementation is economically beneficial, this consensus masks a deeper methodological inconsistency across the literature.

#### 6.1.1. Overemphasis on Maintenance Phase

A striking trend is the dominant focus on the MOL phase. Most positive conclusions stem from studies that assess SHMS benefits in operational contexts—particularly reductions in inspection labor and downtime. This emphasis makes sense, as MOL impacts are tangible and easier to quantify. However, by excluding the BOL stage—where integration costs, sensor placement logistics, and design modifications occur—these studies risk overstating return on investment. The few BOL-inclusive studies offer more tempered or inconclusive findings, suggesting that real-world implementation barriers are being sidelined in optimistic MOL-centric models.

#### 6.1.2. The Weight Paradox

Another revealing contradiction arises from how SHMS weight is modeled. Intuitively, added structural mass should degrade cost-effectiveness by increasing fuel consumption and reducing payload. However, half of the studies that modeled weight still found SHMS to be beneficial, and most of the remaining studies ignored weight altogether. This paradox may reflect overly optimistic assumptions about inspection savings, or it may suggest that when SHMS is integrated early in the design process, weight penalties can be offset through structural optimizations. However, the trend toward more favorable results in studies that omit weight modeling signals a broader issue of optimism bias and incomplete system modeling.

#### 6.1.3. Unrealistic Performance Assumptions

Performance modeling is another weak link in the current literature. Nearly half of the reviewed studies assume perfect SHMS detection (100% POD, 0% PFA), which is incompatible with real-world sensor limitations. Even more concerning is that these idealized studies often report results similar to those that model realistic detection curves—raising questions about model sensitivity. If cost outcomes remain stable despite large differences in assumed system performance, it suggests that models may be insensitive to critical detection variables, undermining their reliability. This oversimplification could result in flawed business cases and misinformed design decisions.

#### 6.1.4. Sensor Type, Aircraft Type, and Structural Focus

Further inconsistencies emerge in the sensor technology and application domain. Most studies focus on piezoelectric sensors, aligning with the 59% of studies targeting metallic structures, where piezo integration is more mature. In contrast, FBG sensors—better suited for composites—are underrepresented, despite the aviation industry’s pivot toward composite airframes. Similarly, rotary-wing aircraft are largely neglected (12%), and e-VTOLs are entirely absent from economic modeling, despite their growing relevance and design flexibility.

#### 6.1.5. Whole-Aircraft vs. Component-Level Monitoring

Nearly half the studies model SHMS at the whole-aircraft level, while the rest target components like fuselage (35%), rotor blades (12%), and wings (6%). While whole-aircraft approaches promise holistic maintenance optimization, they may also involve monitoring low-value areas, diluting cost-effectiveness. Some authors suggest that monitoring inaccessible or fatigue-prone areas (e.g., rotor blades, lower fuselage) delivers higher returns, but this insight is not systematically explored in the literature.

#### 6.1.6. Unaddressed System-Level Impacts

Surprisingly, key system-level burdens—like power consumption, sensor aging, and reliability degradation due to sensor density—are almost entirely neglected. Only 6% of studies address power draw, and the same share consider aging effects, despite known degradation phenomena such as moisture-induced drift in FBGs. Reliability impacts from high sensor density, a well-known concern in certification discussions, are absent from all studies. Without modeling these drawbacks, economic models risk being too detached from operational reality.

#### 6.1.7. Underexplored Benefits

On the other hand, design-enabling benefits—such as safety margin reduction and operational life extension—are also rarely modeled (12% and 6%, respectively). These benefits could significantly amplify SHMS value in clean-sheet aircraft programs, but the lack of modeling suggests that SHMS is still treated as an add-on to legacy platforms rather than a disruptive enabler of structural innovation. This omission limits our understanding of how SHMS could drive next-generation airframe design.

Similarly, SHMS prognostic capabilities, while frequently cited as a long-term goal, have not been economically modeled for structural elements. Some economic studies exist in the context of engine and system prognostics [86,87,88,89], but their insights have not been transferred to airframe applications.

#### 6.1.8. Cost Modeling Variability

Another critical issue is the inconsistency in cost variable inclusion. While maintenance labor is the most frequently modeled cost (13 studies), others—such as sensor integration labor, downtime, ticket revenue, or crew costs—are rarely included. This variability undermines comparability across studies and reflects a lack of standardized economic modeling frameworks. Only one study considered the cost of tickets, a potentially significant variable for commercial operators facing delays or cancellations due to unplanned maintenance.

#### 6.1.9. Broader Implications

These contradictions and omissions point to a field that is still in its exploratory phase, with limited consensus on best practices or necessary modeling rigor. SHMS economic modeling appears to be model-dependent rather than evidence-based, with results heavily influenced by which variables are included and how assumptions are framed.

This reflects broader dynamics in aerospace technology adoption, where innovations like SHMS must overcome certification conservatism, integration cost, and lack of long-term field data. Despite some successful implementations—particularly in military and experimental platforms—widespread civilian adoption remains stalled, not due to technical infeasibility, but because the economic case remains fragmented and context-specific.

### 6.2. Future Directions

While this review identifies clear gaps in the existing literature on the economic evaluation of SHMS, it is equally important to articulate how these gaps can be meaningfully addressed. We propose the following priority areas for future research, along with methodological suggestions for advancing knowledge and practice in each.

- **Quantifying SHMS performance and reliability.**
  SHMS adoption depends on trust in detection performance (POD, PFA), yet economic models often assume perfect performance. Furthermore, none of the studies considered the reliability of the SHMS, which instead may be subjected to faults during the lifecycle. For instance, considering the case study presented in [18], a fuselage equipped with $N_{s}=9988$ piezoelectric sensors was assumed, each with a failure rate $F_{r}=5\%$ over the entire life cycle. The cost to replace failed sensors can be expressed as:
  (1) $$C_{\mathrm{reliability}}=2N_{s}F_{r}t_{\mathrm{install}}\left(C_{\mathrm{mh}}+R_{\mathrm{loss}}\right)K_{\mathrm{unscheduled}}K_{\mathrm{removal}}+F_{r}N_{s}C_{\mathrm{sensor}}$$
  where $C_{\mathrm{mh}}$ is the labor rate, $R_{\mathrm{loss}}$ is the loss of revenue, $t_{\mathrm{install}}$ is the sensors’ installation time during the production of the aircraft, and $C_{\mathrm{sensor}}$ is the cost of one sensor. The factor of 2 accounts for both removal and installation, assuming equal time requirements. According to [66], unscheduled maintenance activities can cost up to five times more than scheduled ones; therefore, a factor $K_{\mathrm{unscheduled}}=5$ was introduced. Furthermore, a factor $K_{\mathrm{removal}}=2.5$ accounts for the additional time needed to remove surrounding structural elements when replacing sensors during service, as $t_{\mathrm{install}}$ reflects only installation during manufacturing. Based on this model, a sensor failure rate of 5% results in an estimated 6% increase in SHMS implementation costs, underscoring the relevance of incorporating reliability effects into future economic assessments. Future works should be aimed at developing probabilistic cost–benefit models that integrate actual or estimated POD/PFA curves, including SHMS failure probability. Studies should simulate outcomes under variable SHMS reliability using Monte Carlo simulations, capturing both false negatives (missed damage) and false positives (unnecessary inspections).
- **Lifecycle Cost Modeling with Integrated Design Phase Consideration**.
  Current studies mostly assess SHMSs during the maintenance phase (MOL), neglecting integration costs and benefits at the design and manufacturing stages (BOL). Researchers should create bottom-up cost models for SHMS integration into airframe design, incorporating manufacturing disruptions, sensor protection needs, and certification costs. Models should include discounted cash flow, net present value, and real-options analysis to evaluate long-term return on investment.
- **Economic Studies for Emerging Aircraft Types.**
  Electrically powered and urban mobility aircraft have distinctive lifecycle profiles that offer opportunities to integrate SHMSs from the outset. As noted by [90], fully exploiting the potential of SHMSs requires its inclusion at the aircraft design stage. In the authors’ view, this presents a particularly valuable opportunity for e-VTOLs, whose architectures are still in development. Future research should therefore focus on conceptual design studies that embed SHMSs early in the e-VTOL and other novel platform architectures, employing multidisciplinary design optimization to account for SHMS-related trade-offs (e.g., weight, power, cost, inspection intervals). A logical first step would be the development of life cycle cost models tailored to e-VTOLs operating under representative mission profiles. Within this context, battery monitoring has received particular attention [91], as lithium-based energy storage systems present fire-related safety risks from thermal runaway, making their health and structural integrity a critical focus of SHMSs.
- **Addressing Operational Costs of False Alarms.**
  False positives drive unnecessary inspections, increasing downtime and labor costs—yet these are rarely modeled. Future works should develop operational simulation models, for instance, discrete-event, that simulate maintenance operations and the economic impact of SHMS-triggered false alarms across a fleet.
- **SHMSs in Probabilistic Design and Safety Margin Reduction**.
  SHMSs may justify reduced structural margins, lowering weight and cost—but few studies quantify this. Research studies should apply probabilistic damage tolerance analysis to evaluate how SHMSs can support the reduction in safety factors without compromising reliability: for instance, assuming a slow growth approach for composite structures could help to consider a safety margin reduction.

### 6.3. Limitations

Although this review offers a structured synthesis of current economic assessments of SHMSs for aerospace structures, several limitations must be acknowledged.

- **Small sample size**: The review includes only 17 studies, reflecting the relative novelty and limited number of publications offering quantitative economic evaluations of SHMSs applied to structural components. This restricts the breadth of comparative analysis and may bias findings toward more frequently studied systems (e.g., piezo-based SHMS on metallic airframes).
- **Scope exclusions**: This review intentionally excludes SHMSs applied to systems-level components (e.g., engines, avionics, hydraulics), despite their significant role in operational failures and maintenance costs. As discussed earlier, our structural focus addresses a specific research gap in economic evaluation, but it does not provide a full picture of SHM applicability across the aircraft lifecycle.
- **Heterogeneity of economic models**: The reviewed studies employ diverse modeling approaches, often with simplified or unreported assumptions. Important factors such as discount rates, sensitivity to failure probabilities, and treatment of uncertainty were not consistently addressed, reducing the ability to generalize findings.

These limitations underscore the need for more standardized, transparent, and comprehensive economic studies in this domain. Our findings should therefore be interpreted as indicative rather than definitive.

## 7. Conclusions

Unlike most literature reviews on SHMSs, this study specifically examines the cost–benefit analyses associated with SHMS applications. A total of seventeen studies were reviewed, with the most relevant features classified to identify general trends and highlight gaps in the current literature. These features include the SHMS type, the material of the monitored structure, the type of vehicle, and the integration of SHMSs into the maintenance process. The cost models presented in each study were also analyzed, and the cost variables were grouped into major categories, including maintenance manpower, fuel consumption, and sensor costs. The analysis revealed that the majority of case studies do not specify the type of SHMS considered, raising concerns about the reliability of a large portion of the reported results. The majority of the studies declaring the type of the SHMSs focus on piezoelectric-based SHMSs and predominantly address metallic fixed-wing aircraft, while only a minority investigate composite structures. Most studies concentrate on the impact of SHMSs during the maintenance phase, with limited attention paid to the integration costs associated with embedding sensors into the structure. In addition, several potential benefits—such as extension of operational life, whose impact was estimated to extend the operational life by 85%, prognostic capabilities, and reduction in structural safety margins—remain largely underexplored, while key drawbacks—such as limited detection performance, false alarms, SHMS reliability, and power consumption—are often overlooked. Notably, no study explicitly addresses SHMS reliability, despite its critical role in lifecycle economic assessments and its importance as a barrier to industrial adoption, indeed, preliminary calculations show that a sensor failure rate of 5% could lead to a 6% increase in the SHMS implementation costs.

Two notable paradoxes were observed: studies accounting for the weight of SHMS did not consistently report a negative economic impact, nor did those assuming realistic SHMS performances. This suggests potential inconsistencies in the underlying cost–benefit modeling.

Among cost variables, maintenance and fuel costs are the most commonly considered, while downtime costs are frequently neglected, despite their significant impact on the economic balance of SHMS systems. While SHMS has the potential to reduce maintenance burdens, the presence of frequent false alarms could offset these benefits, making it essential to develop more accurate models that account for these phenomena. Furthermore, the lack of research on the economic integration of SHMSs into emerging vehicle types, such as e-VTOL aircraft, presents a significant gap. Given the early stages of their design, incorporating SHMS considerations from the outset could lead to significant lifecycle cost reductions.

Future research is essential to address several critical areas. First, models that quantify SHMS reliability, false alarm rates, and the impact of sensor aging and power consumption should be developed to improve the accuracy of economic assessments. Second, more focus is needed on the integration of SHMSs during the manufacturing phase, particularly for composite structures, to better understand associated costs and benefits. Third, the potential advantages of SHMSs, such as extending operational life, reducing safety margins, and enabling prognostics, should be explored and quantified in greater depth. Additionally, the economic implications of downtime costs due to false alarms and maintenance interventions need to be systematically incorporated into cost-benefit analyses. Finally, the design of future aircraft, especially e-VTOLs, provides a unique opportunity to incorporate SHMSs early in the development process, which could significantly reduce lifecycle costs and maximize the economic benefits of SHMS systems.

While existing studies suggest promising economic benefits for SHMSs, further research is necessary to substantiate these findings. A more comprehensive and realistic treatment of SHMS performance and integration factors will be crucial for supporting broader adoption across the aviation industry.

Overall, there is a need for a framework that provides guidelines to standardize the cost–benefit analysis of SHMSs in aviation, ensuring greater consistency across future studies and improving comparability within the existing and future literature.

## Figures and Tables

**Table 1:** Representative literature reviews on structural health monitoring in aerospace applications.

Year	Authors	Title	Focus Area	Citation
2010	Diamanti & Soutis	Structural health monitoring techniques for aircraft composite structures	Damage detection, composites	[22]
2015	Giurgiutiu	SHM of aerospace composites	Technologies, sensors	[1]
2016	Yuan	SHM in aerospace structures	Broad SHM overview	[19]
2020	Güemes et al.	SHM for advanced composite structures: a review	Composites, sensor integration	[21]
2021	Hassani et al.	SHM in composite structures: a comprehensive review	ML, composites	[36]
2025	Kosova et al.	SHM in aviation: future directions for machine learning	ML in SHM	[35]

**Table 2:** Classification of the reviewed literature.

Ref.	Type of Sensor Used in SHMS	Material of the Monitored Structure	Type of Vehicle Monitored	Life Cycle Stages for Economic Assessment	Type of Structural Element Monitored	Integration in Maintenance Process	Impact of SHMS Performance	Impact of SHMS Weight	Impact of SHMS Ageing	Impact of Operational Life Extension	Impact of SHMS Power Consumption	Impact of Safety Margins Reduction	Evaluation of SHMS Cost-Effectiveness
[18]	Piezo	Met.	Fixed Wing	BOL and MOL	Fuselage	Sched.	Perf.	Y	N	N	N	N	Neg.
[58]	Piezo	Comp.	Fixed Wing	BOL and MOL	Fuselage	Sched.	Perf.	Y	N	N	N	N	Param.
[57]	N.S.	Met.	Fixed Wing	MOL	Fuselage	Sched.	Real.	Y	N	N	N	N	Pos.
[56]	N.S.	Met.	Fixed Wing	MOL	Fuselage	Sched., Online, and Hybrid	Real.	Y	N	N	N	N	Pos.
[33]	Piezo	Met.	Fixed Wing	MOL	Whole aircraft	Online	Perf.	Y	N	N	N	N	Param.
[65]	N.S.	Met.	Fixed Wing	MOL	Whole aircraft	Sched.	Perf.	N	N	N	N	N	Neg.
[66]	N.S.	Met.	Fixed Wing	MOL	Whole aircraft	Sched.	Real.	N	N	N	N	N	Pos.
[62]	Piezo and FBG	Met.	Fixed Wing	MOL	Whole aircraft	Online	Perf.	Y	N	N	Y	N	Pos.
[61]	Piezo	Comp.	Fixed Wing	MOL	Whole aircraft	Online	Perf.	Y	N	N	N	Y	Pos.
[67]	N.S.	Comp.	Fixed Wing	MOL	Whole aircraft	Sched. and Online	Real.	N	N	N	N	N	Pos.
[60]	Piezo	Met.	Fixed Wing	MOL	Fuselage	Online	Real.	N	N	N	N	N	Pos.
[59]	Piezo	Comp.	Fixed Wing	MOL	Whole aircraft	Online	Perf.	Y	N	N	N	Y	Pos.
[63]	FBG	Comp.	Rotary Wing	BOL	Rotor blade	N.A.	Perf.	N	N	N	N	N	Param.
[64]	FBG	Comp.	Rotary Wing	MOL	Rotor blade	Sched.	Real.	N	N	N	N	N	Neg.
[69]	N.S.	Met.	Fixed Wing	MOL	Whole aircraft	Usage mon.	Perf.	N	N	Y	N	N	Pos.
[41]	N.S.	Met.	Fixed Wing	BOL and MOL	Fuselage	Sched.	Real.	Y	Y	N	N	N	Pos.
[68]	N.S.	Comp.	Fixed Wing	MOL	Wing	Online	Real.	N	N	N	N	N	Param.

## References

[1] Giurgiutiu V. (2015). Structural Health Monitoring of Aerospace Composites.

[2] Zhu L., Li N.C.P. (2018). Light-weighting in aerospace component and system design. Propuls. Power Res..

[3] European Union Aviation Safety Agency (2023). Acceptable Means of Compliance and Guidance Material to Part 25 (CS-25)—Large Aeroplanes.

[4] Airoldi A., Boiocchi M., Natali M., Mirani C., Di Pancrazio L., Consiglio G., Ballarin P., Riva M. (2023). Feasibility of a morphing rocket nozzle for thrust vector control based on corrugated composite laminates. Appl. Compos. Mater..

[5] Riva M., Airoldi A., Turconi T., Ballarin P., Boiocchi M., Bottasso L. (2023). Development and manufacturing of flexible joints based on corrugated composite laminates. Compos. Struct..

[6] Lightfoot J.S., Wisnom M.R., Potter K. (2013). A new mechanism for the formation of ply wrinkles due to shear between plies. Compos. Part A Appl. Sci. Manuf..

[7] Belnoue J.P.H., Mesogitis T., Nixon-Pearson O.J., Kratz J., Ivanov D.S., Partridge I.K., Potter K.D., Hallett S.R. (2017). Understanding and predicting defect formation in automated fibre placement pre-preg laminates. Compos. Part A Appl. Sci. Manuf..

[8] Tang Z., Hang C., Suo T., Wang Y., Dai L., Zhang Y., Li Y. (2017). Numerical and experimental investigation on hail impact on composite panels. Int. J. Impact Eng..

[9] Juntikka R., Olsson R. Experimental and modelling study of hail impact on composite plates. Proceedings of the 17th International Conference of Composite Materials.

[10] Zhang Y., Zhou Y. (2023). Investigation of bird-strike resistance of composite sandwich curved plates with lattice/foam cores. Thin-Walled Struct..

[11] Georgiadis S., Gunnion A.J., Thomson R.S., Cartwright B.K. (2008). Bird-strike simulation for certification of the Boeing 787 composite moveable trailing edge. Compos. Struct..

[12] Van den Bergh J., De Bruecker P., Beliën J., Peeters J. (2013). Aircraft maintenance operations: State of the art. HUB Res. Pap..

[13] Cho P.Y. (2011). Optimal Scheduling of Fighter Aircraft Maintenance. Ph.D. Thesis.

[14] Steiner A. A heuristic method for aircraft maintenance scheduling under various constraints. Proceedings of the 6th Swiss Transport Research Conference.

[15] Ciampa F., Mahmoodi P., Pinto F., Meo M. (2018). Recent advances in active infrared thermography for non-destructive testing of aerospace components. Sensors.

[16] Bossi R.H., Giurgiutiu V. (2015). Nondestructive testing of damage in aerospace composites. Polymer Composites in the Aerospace Industry.

[17] Oliveira T.L.L., Hadded M., Mimouni S., Schaan R.B. (2025). The Role of Non-Destructive Testing of Composite Materials for Aerospace Applications. NDT.

[18] Dong T., Kim N.H. (2018). Cost-effectiveness of structural health monitoring in fuselage maintenance of the civil aviation industry. Aerospace.

[19] Yuan F.G. (2016). Structural Health Monitoring (SHM) in Aerospace Structures.

[20] Land J.E. (2001). HUMS-the benefits-past, present and future. Proceedings of the IEEE Aerospace Conference Proceedings.

[21] Güemes A., Fernandez-Lopez A., Pozo A.R., Sierra-Pérez J. (2020). Structural health monitoring for advanced composite structures: A review. J. Compos. Sci..

[22] Diamanti K., Soutis C. (2010). Structural health monitoring techniques for aircraft composite structures. Prog. Aerosp. Sci..

[23] Ballarin P., Sala G., Macchi M., Roda I., Baldi A., Airoldi A. (2024). Application of Artificial Neural Networks to a Model of a Helicopter Rotor Blade for Damage Identification in Realistic Load Conditions. Sensors.

[24] Airoldi A., Ballarin P., Di Mauro S., Rigamonti D., Reinert F., Dadras M.M., Zabihzadeh S., De Nicolò E., Bettini P., Cartabia L. Development of an additive manufactured fitting sensorized with optical fibres for load recognition. Proceedings of the AIAA Scitech 2023 Forum.

[25] Staszewski W., Boller C., Tomlinson G.R. (2004). Health Monitoring of Aerospace Structures: Smart Sensor Technologies and Signal Processing.

[26] Wang Y., Hu S., Xiong T., Huang Y., Qiu L. (2022). Recent progress in aircraft smart skin for structural health monitoring. Struct. Health Monit..

[27] Giurgiutiu V. (2015). Structural Health Monitoring (SHM) of Aerospace Composites. Polymer Composites in the Aerospace Industry.

[28] Di Sante R. (2015). Fibre optic sensors for structural health monitoring of aircraft composite structures: Recent advances and applications. Sensors.

[29] Qiu Y., Wang Q.b., Zhao H.t., Chen J.a., Wang Y.y. (2013). Review on composite structural health monitoring based on fiber Bragg grating sensing principle. J. Shanghai Jiaotong Univ. (Sci.).

[30] Falcetelli F., Yue N., Di Sante R., Zarouchas D. (2022). Probability of detection, localization, and sizing: The evolution of reliability metrics in Structural Health Monitoring. Struct. Health Monit..

[31] Wensveen J.G. (2016). Air Transportation: A Management Perspective.

[32] Shaw S. (2016). Airline Marketing and Management.

[33] Cusati V., Corcione S., Memmolo V. (2021). Impact of structural health monitoring on aircraft operating costs by multidisciplinary analysis. Sensors.

[34] Rocha H., Semprimoschnig C., Nunes J.P. (2021). Sensors for process and structural health monitoring of aerospace composites: A review. Eng. Struct..

[35] Kosova F., Altay Ö., Ünver H.Ö. (2025). Structural health monitoring in aviation: A comprehensive review and future directions for machine learning. Nondestruct. Test. Eval..

[36] Hassani S., Mousavi M., Gandomi A.H. (2021). Structural health monitoring in composite structures: A comprehensive review. Sensors.

[37] Reveley M.S., Briggs J.L., Evans J.K., Jones S.M., Kurtoglu T., Leone K.M., Sandifer C.E. (2011). Causal Factors and Adverse Events of Aviation Accidents and Incidents Related to Integrated Vehicle Health Management.

[38] Sbarufatti C., Manes A., Giglio M. (2013). Performance optimization of a diagnostic system based upon a simulated strain field for fatigue damage characterization. Mech. Syst. Signal Process..

[39] Sala G., Di Landro L., Airoldi A., Bettini P. (2015). Fibre optics health monitoring for aeronautical applications. Meccanica.

[40] Verhagen W.J., Santos B.F., Freeman F., van Kessel P., Zarouchas D., Loutas T., Yeun R.C., Heiets I. (2023). Condition-Based Maintenance in Aviation: Challenges and Opportunities. Aerospace.

[41] Pattabhiraman S., Kim N.H., Haftka R. Effects of uncertainty reduction measures by structural health monitoring on safety and lifecycle costs of aircrafts. Proceedings of the 51st AIAA/ASME/ASCE/AHS/ASC Structures, Structural Dynamics, and Materials Conference 18th.

[42] Air Transport Association of America (2007). ATA MSG-3: Operator/Manufacturer Scheduled Maintenance Development.

[43] Ahmadi A., Söderholm P., Kumar U. (2010). On aircraft scheduled maintenance program development. J. Qual. Maint. Eng..

[44] Rajamani R. (2018). Condition-Based Maintenance in Aviation: The History, the Business and the Technology.

[45] Janapati V., Kopsaftopoulos F., Li F., Lee S.J., Chang F.K. (2016). Damage detection sensitivity characterization of acousto-ultrasound-based structural health monitoring techniques. Struct. Health Monit..

[46] Kaplan M.P., Lincoln J.W. (1996). The US Air Force approach to aircraft damage tolerant design. Fatigue and Fracture.

[47] Garg A.C. (1988). Delamination—A damage mode in composite structures. Eng. Fract. Mech..

[48] Wang S. (1983). Fracture mechanics for delamination problems in composite materials. J. Compos. Mater..

[49] Federal Aviation Administration (2009). AC 20-107B: Composite Aircraft Structure.

[50] Andrade P., Silva C., Ribeiro B., Santos B.F. (2021). Aircraft maintenance check scheduling using reinforcement learning. Aerospace.

[51] Deng Q., Santos B.F. (2022). Lookahead approximate dynamic programming for stochastic aircraft maintenance check scheduling optimization. Eur. J. Oper. Res..

[52] Galanopoulos G., Eleftheroglou N., Milanoski D., Broer A., Zarouchas D., Loutas T. (2023). A novel strain-based health indicator for the remaining useful life estimation of degrading composite structures. Compos. Struct..

[53] Galanopoulos G., Milanoski D., Broer A.A., Zarouchas D., Loutas T. (2021). Health indicators for diagnostics and prognostics of composite aerospace structures. Proceedings of the 2021 IEEE 8th International Workshop on Metrology for AeroSpace (MetroAeroSpace).

[54] Cristiani D., Sbarufatti C., Cadini F., Giglio M. (2021). Fatigue damage diagnosis and prognosis of an aeronautical structure based on surrogate modelling and particle filter. Struct. Health Monit..

[55] Sprong J.P., Jiang X., Polinder H. (2020). Deployment of Prognostics to Optimize Aircraft Maintenance—A Literature Review. J. Int. Bus. Res. Mark..

[56] Pattabhiraman S., Gogu C., Kim N.H., Haftka R.T., Bes C. (2012). Skipping unnecessary structural airframe maintenance using an on-board structural health monitoring system. Proc. Inst. Mech. Eng. Part O J. Risk Reliab..

[57] Pattabhiraman S., Kim N.H., Haftka R. Effect of inspection strategies on the weight and lifecycle cost of airplanes. Proceedings of the 52nd AIAA/ASME/ASCE/AHS/ASC Structures, Structural Dynamics and Materials Conference.

[58] Giannakeas I.N., Khodaei Z.S., Aliabadi M.F. (2022). Structural health monitoring cost estimation of a piezosensorized aircraft fuselage. Sensors.

[59] Cusati V., Corcione S., Memmolo V. (2022). Potential benefit of structural health monitoring system on civil jet aircraft. Sensors.

[60] Fitzwater L., Davis C., Torng T., Poblete J. Cost/benefit analysis for intergration of non-deterministic analysis and in-situ monitoring for structural integrity. Proceedings of the 52nd AIAA/ASME/ASCE/AHS/ASC Structures, Structural Dynamics and Materials Conference.

[61] Dienel C.P., Meyer H., Werwer M., Willberg C. (2019). Estimation of airframe weight reduction by integration of piezoelectric and guided wave–based structural health monitoring. Struct. Health Monit..

[62] Büchter K.D., Sebastia Saez C., Steinweg D. (2022). Modeling of an aircraft structural health monitoring sensor network for operational impact assessment. Struct. Health Monit..

[63] Ballarin P., Macchi M., Roda I., Sala G., Baldi A., Airoldi A. (2024). Economic Impact Assessment of Structural Health Monitoring Systems on Helicopter Blade Beginning of Life. Struct. Control Health Monit..

[64] Ballarin P., Macchi M., Roda I., Sala G., Baldi A., Airoldi A. (2024). Economic Impact Assessment of Structural Health Monitoring Systems on the Lifecycle of a Helicopter Blade. e-J. Nondestruct. Test..

[65] Kapoor H., Braun C., Boller C. Modelling and optimisation of maintenance intervals to realise Structural Health Monitoring applications on aircraft. Proceedings of the 5th European Workshop on Structural Health Monitoring.

[66] Sun J., Chen D., Li C., Yan H. (2018). Integration of scheduled structural health monitoring with airline maintenance program based on risk analysis. Proc. Inst. Mech. Eng. Part O J. Risk Reliab..

[67] Chen X., Bil C., Ren H. Influence of SHM techniques on scheduled maintenance for aircraft composite structures. Proceedings of the 14th AIAA Aviation Technology, Integration, and Operations Conference.

[68] Cottone G., Gollwitzer S., Heckenberger U., Straub D. Reliability-oriented optimization of replacement strategies for monitored composite panels for aircraft structures. Proceedings of the 9th International Workshop on Structural Health Monitoring.

[69] Pfingstl S., Steinweg D., Zimmermann M., Hornung M. (2022). On the potential of extending aircraft service time using load monitoring. J. Aircr..

[70] Giurgiutiu V. (2007). Structural Health Monitoring: With Piezoelectric Wafer Active Sensors.

[71] Jiao P., Egbe K.J.I., Xie Y., Matin Nazar A., Alavi A.H. (2020). Piezoelectric sensing techniques in structural health monitoring: A state-of-the-art review. Sensors.

[72] Qing X., Li W., Wang Y., Sun H. (2019). Piezoelectric transducer-based structural health monitoring for aircraft applications. Sensors.

[73] Takeda N., Okabe Y., Kuwahara J., Kojima S., Ogisu T. (2005). Development of smart composite structures with small-diameter fiber Bragg grating sensors for damage detection: Quantitative evaluation of delamination length in CFRP laminates using Lamb wave sensing. Compos. Sci. Technol..

[74] Grattan K.T., Sun T. (2000). Fiber optic sensor technology: An overview. Sens. Actuators A Phys..

[75] Ramakrishnan M., Rajan G., Semenova Y., Farrell G. (2016). Overview of fiber optic sensor technologies for strain/temperature sensing applications in composite materials. Sensors.

[76] Stehmeier H., Speckmann H. (2004). Comparative vacuum monitoring (CVM). Proceedings of the 2nd European Workshop on Structural Health Monitoring.

[77] Roach D. (2009). Real time crack detection using mountable comparative vacuum monitoring sensors. Smart Struct. Syst..

[78] Blond K., O’Brien T., Thompson N., Piotrowski D., Clark A. (2023). Comparative Vacuum Monitoring Solutions to Advance US Air Force KC-46A Condition-Based Maintenance Plus. Aerospace.

[79] Ouertani M.Z., Parlikad A.K., McFarlane D.C. (2008). Towards an approach to Select an Asset Information Management Strategy. Int. J. Comput. Sci. Appl..

[80] Badurdeen F., Shuaib M., Liyanage J.P. (2012). Risk modeling and analysis for sustainable asset management. Proceedings of the Engineering Asset Management and Infrastructure Sustainability: Proceedings of the 5th World Congress on Engineering Asset Management (WCEAM 2010).

[81] Kiritsis D., Nguyen V.K., Stark J. (2008). How closed-loop PLM improves Knowledge Management over the complete product lifecycle and enables the factory of the future. Int. J. Prod. Lifecycle Manag..

[82] Mendler A., Döhler M., Grosse C.U. (2024). Predictive probability of detection curves based on data from undamaged structures. Struct. Health Monit..

[83] Aceti P., Sala G. (2024). Impact of Moisture Absorption on Optical Fiber Sensors: New Bragg Law Formulation for Monitoring Composite Structures. J. Compos. Sci..

[84] Aceti P., Calervo L., Bettini P., Sala G. (2025). Measurement and Decoupling of Hygrothermal-Mechanical Effects with Optical Fibers: Development of a New Fiber Bragg Grating Sensor. Sensors.

[85] Baker W., McKenzie I., Jones R. (2004). Development of life extension strategies for Australian military aircraft, using structural health monitoring of composite repairs and joints. Compos. Struct..

[86] Hölzel N.B., Schilling T., Gollnick V. An aircraft lifecycle approach for the cost-benefit analysis of prognostics and condition-based maintenance based on discrete-event simulation. Proceedings of the Annual Conference of the PHM Society.

[87] Vandawaker R.M., Jacques D.R., Freels J.K. (2015). Impact of prognostic uncertainty in system health monitoring. Int. J. Progn. Health Manag..

[88] Vandawaker R.M., Jacques D.R., Ryan E.T., Huscroft J.R., Freels J.K. (2017). Health monitoring impact on non-repairable component supply methods. J. Qual. Maint. Eng..

[89] Omoleye T.J., Alabdulkarim A.A., Tsui K.L. (2019). Impact of resources and monitoring effectiveness on prognostics enabled condition based maintenance policy. J. Simul..

[90] Boller C. (2000). Next generation structural health monitoring and its integration into aircraft design. Int. J. Syst. Sci..

[91] Granado L., Ben-Marzouk M., Saenz E.S., Boukal Y., Jugé S. (2022). Machine learning predictions of lithium-ion battery state-of-health for eVTOL applications. J. Power Sources.

